# Supplementary material for: Swine influenza A virus isolates containing the pandemic H1N1 origin matrix gene elicit greater disease in the murine model
Source: Microbiol Spectr. 2024 Feb 1;12(3):e03386-23. doi: 10.1128/spectrum.03386-23 (PMC10913740; doi:10.1128/spectrum.03386-23)
Supplement: Supplemental material — Tables S1 and S2; Figure S1. [file spectrum.03386-23-s0001.docx]

| **Influenza Strain** | | **NCBI Taxon ID** | **Accession Numbers** | | | | | | | |
| --- | --- | --- | --- | --- | --- | --- | --- | --- | --- | --- |
|  |  |  | **PB2** | **PB1** | **PA** | **HA** | **NP** | **NA** | **M** | **NS** |
| A/CA/07/09 (H1N1) | CA/07 | NCBI:txid641809 |  | | | | | | | |
| A/swine/Missouri/A01444664/2013 (H1N2) | MO/664/swM |  | MW656331 | MW656333 | MW656334 | MW656335 | MW656337 | MW656330 | MW656332 | MW656336 |
| A/sw/North Carolina/A01394568/2013 (H1N1) | NC/568/pdmM |  | MW656355 | MW656358 | MW656360 | MW656361 | MW656359 | MW656354 | MW656357 | MW656356 |
| A/swine/North Carolina/152702/2015 (H1N2) | NC/702/pdmM |  | MW656319 | MW656318 | MW656321 | MW656316 | MW656320 | MW656317 | MW656322 | MW656315 |
| A/swine/North Carolina/154074/2015 (H1N1) | NC/074/pdmM |  | MW656305 | MW656309 | MW656306 | MW656303 | MW656307 | MW656304 | MW656308 | MW656302 |
| A/sw/Illinois/A00857300/2011 (H1N1) | IL/300/swM |  | MW656347 | MW656346 | MW656348 | MW656352 | MW656351 | MW656353 | MW656349 | MW656350 |
| A/sw/Indiana/A00968351/2011 (H1N1) | IN/351/swM |  | MW656338 | MW656344 | MW656340 | MW656343 | MW656339 | MW656345 | MW656341 | MW656342 |
| A/sw/Minnesota/A01125993/2012 (H3N2) | MN/993/swM |  | MW656323 | MW656325 | MW656328 | MW656324 | MW656329 | MW656326 | OR553107 | MW656327 |
| A/sw/North Carolina/157671/2015 (H3N2) | NC/671/pdmM |  | OR553108 | OR553109 | OR553110 | MW656314 | MW656311 | MW656310 | MW656312 | MW656313 |

**S. Table 1. NCBI taxonomy ID and GenBank accession numbers for the virus strains used in this study.**

| **Influenza Strain** | | **PB2** | **PB1** | **PA** | **HA** | **NP** | **NA** | **M1**  **M2** | **NS1**  **NEP** |
| --- | --- | --- | --- | --- | --- | --- | --- | --- | --- |
| A/swine/Missouri/A01444664/2013 (H1N2) | MO/664/swM | 99.1 | 99.2 | 99.0 | 90.8 | 97.2 | 89.1 | 94.8  85.6 | 95.4  96.7 |
| A/swine/North Carolina/152702/2015 (H1N2) | NC/702/pdmM |  |  |  |  |  |  |  |  |
| **Influenza Strain** | | **PB2** | **PB1** | **PA** | **HA** | **NP** | **NA** | **M1**  **M2** | **NS1**  **NEP** |
| A/swine/Indiana/A00968351/2011 (H1N1) | IN/351/ swM | 99.1 | 100.0 | 99.4 | 97.7 | 97.2 | 96.8 | 94.0  85.6 | 96.3  99.2 |
| A/swine/North Carolina/154074/2015 (H1N1) | NC/074/pdmM |  |  |  |  |  |  |  |  |
| **Influenza Strain** | | **PB2** | **PB1** | **PA** | **HA** | **NP** | **NA** | **M1**  **M2** | **NS1**  **NEP** |
| A/swine/Illinois/A00857300/2011 (H1N1) | IL/300/ swM | 98.9 | 98.5 | 97.2 | 97.7 | 97.8 | 96.8 | 94.4  88.7 | 95.4  97.5 |
| A/swine/North Carolina/A01394568/2013 (H1N1) | NC/568/pdmM |  |  |  |  |  |  |  |  |
| **Influenza Strain** | | **PB2** | **PB1** | **PA** | **HA** | **NP** | **NA** | **M1**  **M2** | **NS1**  **NEP** |
| A/swine/Minnesota/A01125993/2012 (H3N2) | MN/993/swM | 97.8 | 99.3 | 98.4 | 94.2 | 97.8 | 96.2 | 94.4  86.5 | 96.7  94.5 |
| A/swine/North Carolina/157671/2015 (H3N2) | NC/671/pdmM |  |  |  |  |  |  |  |  |

**S. Table 2. Percent homology based on predicted amino acid sequence comparison between matching gene segments**

**Supplemental Figure 1. Histological lesion scores from DBA/2 mice infected with swine influenza viruses.** DBA/2 mice were inoculated with 1e5 pfu of the indicated viruses, euthanized, and lungs fixed for histological analysis at 2 and 4 DPI (n = 2 mice/virus/collection, repeated). Histologic lesion scores out of 22 for DBA/2 mice inoculated with either (A) H1 or (B) H3 swine influenza isolates. Statistical differences were calculated using two-way ANOVA between (A) MO/664/swM and the other viruses with Dunnett post-hoc test or (B) MN/993/swM and NC/671/pdmM with Bonferroni post-hoc test. * <0.05, **<0.005, *** <0.001, **** <0.0001.
